# Supplementary material for: Are carbon emissions trading and green financial instruments synergistic? -Comprehensive quantitative research based on content analysis
Source: PLoS One. 2024 Mar 7;19(3):e0298601. doi: 10.1371/journal.pone.0298601 (PMC10919601; doi:10.1371/journal.pone.0298601)
Supplement: S1 Data — (ZIP) [file pone.0298601.s001.zip › Data/地级市Z_F工作报告.zip!/║╙▒▒/╨╧╠¿╩╨/└┤╫╘╣1⁄2╓┌║┼í╛┬φ┐╦╩2╛▌═°í┐_2008.docx]

各位代表：

　　现在，我代表邢台市人民政府，向大会报告过去五年的工作，对今年工作提出建议，请予审议。并请市政协各位委员和列席会议的同志提出意见。

　　一、过去五年的回顾

　　市十二届人大一次会议以来的五年，是我市改革发展进程中不平凡的五年。全市人民在省委、省政府和市委的正确领导下，以邓小平理论和“三个代表”重要思想为指导，全面落实科学发展观，解放思想，齐心协力，开拓创新，真抓实干，努力克服各种困难和风险，经济社会事业的发展呈现出速度加快、后劲增强、效益提高、民生改善的良好态势，为下一步发展奠定了坚实基础。

　　（一）综合经济实力明显增强。2007年全市实现生产总值890亿元，比2002年增长79.4%，年均增长12.4%。全部财政收入完成85.1亿元，其中地方一般预算收入达31.4亿元，是2002年的2.44倍，年均增长19.5%。县域经济加快发展，2007年全部财政收入过亿元的县（市）达11个，比2002年增加5个，一些基础较差、实力较弱的县实现了跨越式发展。我们把加快工业化进程作为实现富民强县兴市目标的战略重点，紧紧抓住项目建设这个龙头，围绕改造提升传统产业、发展壮大新兴产业，连续实施了项目建设年、工业突破年活动，全市项目建设和产业升级呈现出前所未有的好势头。五年累计完成全社会固定资产投资1772亿元，其中，城镇固定资产投资累计完成1057亿元。一批投资超过10亿元的重大产业支撑项目顺利实施，邢钢精品钢技改、德龙技改、金能东庞矿技改等建成投产；中钢邢机技改、建滔化工、沙河迎新浮法玻璃等项目进展顺利；旭阳煤化工、晶龙集团光伏发电项目被列为河北省重大产业支撑项目。2007年，全市规模以上工业完成增加值294亿元，比2002年翻了一番半。我们大力发展培育有比较优势的新兴产业，实行传统特色产业的升级改造，实施骨干龙头企业再造工程，煤化工、冶金机械装备制造、硅材料、纺织服装、食品医药、新型建材等六大产业在全市工业经济中的主导作用日益突出，占全部工业的比重达到60%以上；一批骨干企业在资本运营、技术改造、经营管理等方面加大了力度，实力和竞争力不断增强，规模以上工业企业达到近900家，销售收入超10亿元的企业达到14家，其中近百亿元的企业4家；五年来，新增中国驰名商标2个，中国名牌产品3项，国家免检产品15项。新型特色产业园区已经成为集群集约、科学发展的有效载体，煤化工基地、线材制品加工基地和宁晋、清河、沙河、隆尧、临西、平乡、南宫等县（市）的特色产业园区不断发展壮大，巨鹿、广宗、任县、南和、新河等县的特色产业园区建设初具规模。我们把优化结构、集群集约、节能减排作为工业发展的主要方式，五年来，淘汰落后炼铁产能15万吨、小水泥产能90万吨、关停小火电机组56.6万千瓦、小玻璃产能1800万重量箱；关停不符合安全生产条件的地方煤矿矿井72对，非煤矿山696家，整合非煤矿山315家。我们加大了服务业发展推进力度，出台了一系列支持引导政策，服务业活力不断增强，2007年完成增加值240亿元，比2002年增长82.1%，年均增长12.7%，商贸流通、餐饮等传统服务业有了新提高，现代物流、旅游、金融、房地产、信息服务等现代服务业有了新发展。

　　（二）新农村建设扎实推进。我们认真落实国家支农惠农政策，农业税、特产税全部取消，累计发放粮食直补、农机具补贴等政策性补贴7.8亿元。积极调整农业结构，2007年全市粮食总产394万吨，比2002年增长11.7%；畜牧、蔬菜、林果三大主导产业占大农业的比重比2002年提高11个百分点；农林复合经营面积累计完成70万亩。大力发展农业产业化经营，市级农业产业化龙头企业达到126家，比2002年增96家，千喜鹤公司成为北京奥运会冷鲜猪肉及肉制品独家供应商。农业综合开发土地治理项目完成资金2.8亿元，新增农业综合生产能力近9000万公斤。农业生态建设稳步推进，太行山绿化工程完成人工造林6.4万亩，黄河故道沙化土地综合治理工程完成造林9.4万亩。进一步加大扶贫投入力度，五年来初步解决了12万贫困人口的温饱问题。农村基础设施建设加快，投资20亿元，新改建农村公路6300多公里，全部实现了村村通公路目标；投资11亿元对农村电网进行了改造；黑龙港流域大力实施联村集中供水工程，先后投资2.8亿元，解决了72万人的饮水安全问题；文明生态村建设取得明显成效，五年来累计投入资金6.5亿元，改造建设1559个行政村，农田水利、广播电视、通信网络等得到完善，农民群众的生产生活条件不断改善。

　　（三）城镇化进程显著加快。2007年全市城镇化水平达38%，比2002年提高8.8个百分点。按照城乡统筹的理念，合理布局城镇体系，促进产业、人口向城镇集中，坚持高起点规划、高标准建设、高质量管理，编制了邢台市城市总体规划，完成了城市道路综合交通、绿地系统、历史文化名城保护等专项规划，内邱、威县、临城、柏乡、任县等县的县城总体规划编制完成。围绕打造宜居、宜业城镇，完善功能，提升品位，加大了城镇建设力度。五年来，中心城市投资113亿元，完成了中兴大街改造、泉北大街建设、泉南大街东延和污水处理厂等一批基础设施重点工程建设。投资12.9亿元，启动了七里河综合治理项目，完成了钢铁路大桥、新华路大桥、东三环大桥新（扩）建等工程。各县城和重点镇也都重视了规划编制，围绕拉大框架，健全基础设施和完善功能分区，谋划建设了一批精品工程，吸纳要素和辐射带动能力进一步增强。城市管理水平有了新提高，市区市容环境卫生整治、拆墙透绿、出入市口整治等活动初见成效，2007年拆违拆迁120万平方米；城区绿化覆盖率达到37%，人均公共绿地面积达到8.9平方米；市区空气质量明显好转，2007年二级及好于二级天数达317天，较2002年增加了153天；我市成功申报为省级历史文化名城，市区和清河被省政府批准为省级园林城市（县城）。交通建设实现新突破，累计完成投资120多亿元，全市公路通车总里程达13058公里，自主建设的邢临高速公路竣工通车。

　　（四）改革开放不断深化。县属国有企业改革基本完成，市属43家国有工业企业实行了股份制等多种形式的产权制度改革，邢机、轮胎厂等一批骨干企业通过改制重组焕发了生机。民营经济增加值占全市GDP的比重达80.5%，上缴税金占全市财政收入的比重达51.5%。成立了市行政服务中心，40个部门和10个中介服务机构的近500项审批和服务事项，统一集中到中心办理，减少了审批环节，提高了工作效率。对外开放水平不断提高，五年实际利用外资7.2亿美元，年均增长39.6%；引进市外资金265.9亿元，年均增长22.2%。中钢、中煤、中盐、香港建滔、日本松宫、美国卡博特等一批国内外知名企业先后到我市投资设厂。与深交所签订了合作推进我市企业上市协议，晶澳太阳能、德龙钢铁等企业在境外成功上市，城市信用社重组升级为市商业银行。对外贸易快速发展，五年来外贸进出口总量完成36亿美元，年均增长25.2%。

　　（五）社会事业协调发展。群众收入有了明显增加，2007年城镇居民人均可支配收入达10575元，农民人均纯收入达3879元，分别比2002年增加4702元和1286元。积极扩大就业再就业，五年来向非农产业转移农村劳动力110万人（次），城镇登记失业率低于省控目标。社会保险和社会救助覆盖面扩大，五年累计支付各项社保待遇40多亿元。农村低保开始启动。城镇低保实现了动态管理下的应保尽保，对低收入困难家庭实行了廉租房和经济适用房货币补贴政策。科技创新能力不断增强，多家企业与高等院校和科研单位联合建立了产学研合作基地，攻克了一批关键技术难题，取得国内先进水平以上的科技成果300项。节能减排成效显著，五年累计投资20亿元，推广了9大重点节能工程，实施了120个节能、环保项目。积极推进教育均衡发展，认真落实“两免一补”政策；全市九年义务教育和学前三年教育得到全面普及，高中毛入学率达到68%；职业技术教育取得新成就，邢台职业技术学院被列入全国职业院校“211工程”，技师学院被国家定为农民工培训示范基地；高等教育跨上新台阶，邢台师专成功升格为本科院校，获得学士学位授予权。文化事业发展步伐加快，投资6000万元加强了公共文化设施建设，推出了一批在省内外有影响的精品节目。医疗卫生事业全面发展，抗击“非典”工作成效显著，疾病预防与控制体系建设基本完成，重大传染病防控能力明显提高；农村三级医疗卫生服务网络建设加快，城市社区卫生服务网络基本形成，城镇医疗水平不断提高；全市425万农民参加新型农村合作医疗，参合率达76%。人才队伍进一步壮大，共引进各类人才2万余人。支持发展残疾人事业，成功创建全国首个“零缺肢市”。人口和计划生育工作初步扭转了被动局面。群众体育活动蓬勃开展，竞技体育水平不断提高。安全生产工作加大力度，遏制了事故频发的势头，实现了总体稳定、趋向好转的良好态势。“双拥”工作实现新突破。精神文明建设成效显著。严厉打击了各类刑事犯罪，社会治安防控体系得到全面加强，平安邢台建设取得初步成效。民族宗教、审计、统计、人防、气象、档案、地方志等工作也都取得了新成绩。

　　五年来，我们始终重视政府自身改革和建设，科学民主决策、依法行政和行政监督的水平有了新提高。市政府自觉接受人大的法律监督、工作监督和政协的民主监督，定期向人大报告工作，向政协通报情况，主动听取人大代表、政协委员的意见和建议，办理人大代表建议、批评和意见1112件，政协提案2543件，办复率达到100%。认真做好信访工作，集中解决了一批群众反映强烈的突出问题。深入开展廉政建设和反腐败斗争，积极实施依法行政，加强执法监督、法律宣传和教育，加快民主法治进程，努力推进法治政府、责任政府、服务型政府建设。

　　各位代表！

　　过去的五年，是改革开放以来我市经济综合发展态势最好的时期之一，是各项社会事业发展最快的时期之一，是人民群众得到实惠最多的时期之一。这些成绩的取得，得益于省委、省政府和市委的正确领导，得益于市人大、市政协和各民主党派的监督支持，得益于全市人民的团结奋斗、开拓进取。在此，我代表邢台市人民政府，向全市广大工人、农民、知识分子、各级干部和社会各阶层的建设者，向人大代表、政协委员，向各民主党派、工商联、无党派人士和人民团体，向驻邢部队、武警官兵和政法干警，向中省直驻邢各单位，向关心和支持邢台发展的海内外朋友，致以崇高的敬意和衷心的感谢！

　　过去的五年，我们在发展实践中积累经验，得到了许多有益的启示：

　　一是必须不断解放思想。解放思想是我们克服经验主义、惯性思维，促进经济社会又好又快发展的一大法宝。我们坚持解放思想、实事求是、与时俱进，不断深化对邢台市情和经济社会发展规律的认识，准确把握所处的发展阶段和方位，谋划并不断完善了全市总体发展思路和一系列重大举措。积极主动应对不断变化的新形势，有效破解发展中的瓶颈和难题，创造性地推进了各项工作。

　　二是必须全面落实科学发展观。科学发展观是我们改变落后面貌、赶超先进的最佳捷径、唯一选择。我们坚持以人为本，把加快发展作为第一要务，坚持突出重点，注重统筹兼顾，推动全面协调可持续发展，着力转变经济发展方式，调整经济结构，促进产业升级，狠抓节能减排，促进了经济发展的速度、质量、结构、效益的同步提高和改善。

　　三是必须深入推进改革开放。改革是推进发展的根本动力，开放是改变落后的必由之路。我们注重用市场经济的思路、理念和办法破解发展中的瓶颈和难题，深入推进国有企业、行政管理体制等改革，增强了经济社会发展活力。不断加大开放步伐，提高开放水平，吸引了国内外一批大企业、大集团来我市投资创业，有力支持了我市经济的发展。

　　四是必须强力优化发展环境。抓发展必须抓环境。我们以转变政府职能为切入点，健全优化环境工作体制，完善行政审批制度，坚持依法行政，推进政务公开，积极打造阳光政府、效率邢台，政府工作透明度不断增强，行政效率进一步提高，服务质量进一步改善。

　　五是必须高度关注民生。不断改善和提高全市人民的生活水平是政府工作的本质要求。我们把实现好、维护好、发展好最广大人民的根本利益作为政府工作的出发点和落脚点，高度重视以民生为重点的社会建设，从增加群众收入、提高公共服务能力和质量入手，着力解决群众上学难、就业难、看病难、住房难等问题，使全市人民共享改革发展成果。

　　我们也清醒地认识到，我市经济社会发展和政府工作中还存在不少矛盾和问题。主要表现：一是综合经济实力弱。与全省平均水平和周边地市相比，我市经济总量小，发展速度慢，产业层次低，质量和效益不高，地方财力不足；城镇化水平低，三产发展滞后；全民创业氛围不浓，市场主体少，活力不足。二是经济发展方式仍比较粗放。主导产业链条短，科技含量高、附加值高的产品少，立市立县的大项目、好项目少；自主创新能力不强，新型工业化步伐不快，经济增长的资源环境代价过大，节能减排和环境保护形势严峻；总量偏小和结构不优的问题同时存在。三是民生和社会事业欠账较多。城镇居民收入和农民收入在全省处于后进位次；文化、教育、体育、卫生等基础设施落后而且发挥作用不够；人才密度小，人力资源结构不优问题较为突出；安全生产基础薄弱，一些安全隐患依然存在。四是一些政府部门和个别工作人员服务意识差、工作节奏慢、办事效率低，改革创新意识不强，推诿扯皮、吃拿卡要等现象依然存在；执行力不强，中梗阻等现象时有发生，等等。对此我们一定保持清醒认识，增强紧迫感、危机感和责任感，采取有效措施，认真加以解决。

　　二、2008年主要任务

　　2008年是落实十七大精神的第一年，也是实施“十一五”规划的关键一年，做好全年工作意义十分重大。政府工作的总体要求是：深入贯彻落实党的十七大和省委、市委七届三次全会精神，进一步解放思想，全面落实科学发展观，深入实施“1346”发展思路，坚持好字优先，好中求快、好中求大、好中求强，以项目建设为龙头，全力加快工业化进程，突出发展第三产业，努力提高城镇化水平，扎实推进新农村建设，着力改善民生，为全面建设小康社会奠定坚实基础。

　　今年全市经济社会发展的主要预期目标是：在优化结构、提高效益、降低消耗、保护环境的基础上，全市生产总值增长13%以上，超过1000亿元；全部财政收入增长16%以上，超过100亿元，其中地方一般预算收入增长20%以上；全社会固定资产投资增长20%以上；规模以上工业增加值增长20%以上，实现利税增长25%以上；服务业增加值增长13%以上，社会消费品零售总额完成317亿元；万元GDP能耗下降4.6%以上；主要污染物COD和二氧化硫排放分别下降4.5%和5.5%；城镇居民人均可支配收入增长11%以上，农民人均纯收入增长6%以上；城镇新增就业4万人，登记失业率控制在4%以内。

　　今年国家将实行稳健的财政政策和从紧的货币政策，目前国际国内经济发展面临诸多不利因素和不确定因素，这对我们做好经济工作提出了新的更高要求。我们要从邢台实际出发，顺时应势，积极应对，坚持发挥优势，遵循规律，抢抓机遇，开放创新，在调整转变中加快发展。重点抓好以下八个方面的工作：

　　（一）加大项目建设力度，促进产业优化升级。投资是我市经济结构优化调整和平稳较快发展的主要动力，我们要继续加大投入力度，正确引导资金投向，努力保持投资的合理较快增长，积极谋划建设一批投资规模大、资源消耗少、科技含量高、环境污染小、产业关联度高、带动性强的大项目，进一步加快工业化进程，增强经济发展后劲，提升区域经济的实力、活力、竞争力。

　　继续开展“项目建设年”活动，加强对项目建设的跟踪服务，确保61个新项目按期开工，49个续建项目加快进度，70个计划竣工项目顺利投产。重点推进沙河2×60万千瓦电厂、金能30万吨钾碱、6万吨玻纤、中钢邢机技改扩能、卡博特30万吨炭黑、建滔50万吨醋酸、旭阳煤化深加工、德龙精品轧辊等总投资10亿元以上的项目建设。加快重大项目的前期工作进度，重点做好邢钢30万吨不锈钢、建滔180万吨大甲醇等重大工业项目前期工作，力争早日开工。要特别重视项目谋划，围绕煤化工、硅材料、钢制品、自行车、轴承、电缆、羊绒制品、纺织服装、新型玻璃等产业谋划一批投入大、效益好、带动性强的项目，确保项目投入的持续增加，促进产业做大做优做强；积极谋划和发展一批包装、运输、配件等关联度高、配套性强的项目，增强产业整体竞争力。要积极推进交通、电力等基础设施项目建设，今年争取邢汾高速公路开工建设，为大广高速公路建设做好配套服务。继续完善电网建设，投资力争超过10亿元。要提高项目建设质量，在项目投资规模、投资强度、建筑密度、节能减排等方面明确具体要求，对重点项目采取分类管理，重点支持发展一批投资规模大、市场前景好、技术设备先进、节能环保的优质项目。对总投资超亿元的54个建设项目搞好服务。

　　坚持以市场为导向，以技术创新为先导，发展有比较优势的主导产业，延伸产业链条，引进和培植上下游产业，注重产业链之间的关联、协作与分工，形成产业群，走集群发展之路，推进产业结构优化升级。一是新兴产业规模化。煤化工、冶金机械装备制造、硅材料三大新兴产业，要靠扩大规模增强实力，靠延长产业链条扩大优势，靠集群集约增强竞争力，到年底增加值增长30%以上。金能、旭阳、建滔、龙星等煤化工企业要突出抓好煤炭深加工增值，延长产业链，提升价值链，做好煤焦油、炭黑、苯加氢、甲醇、醋酸深加工等重点项目，尽快形成煤化工基地。邢钢、德龙、龙海、吉泰等钢铁企业要重点发展精品钢、特种钢和板带线材深加工等，尽快形成钢制品深加工基地；加快中钢邢机技改和高端精品轧辊项目建设。支持晶龙集团加速扩张，以单晶硅向太阳能电池升级为主线，加快建设宁晋和高开区两大硅材料生产和深加工基地。二是传统产业新型化。对纺织服装、食品医药、新型建材三大产业，要运用先进适用技术进行改造提升，引进和培育骨干龙头企业，带动产业升级，到年底，增加值增长20%以上。宁晋、南宫、巨鹿等纺织企业集中地区要进一步调整优化产业布局，促进产品档次、技术工艺和产业集中度的提升。发挥今麦郎、燕南等食品加工企业的龙头带动作用，大力开发方便、绿色、环保、安全的食品和饮品；促进恒利、复星、恒祥、东盛英华等医药生产和流通企业加快发展。鼓励奎山、金牛等水泥企业发展新型干法水泥，鼓励晶牛集团、迎新集团等大力发展优质浮法玻璃，提升建材行业的产品科技含量和附加值。三是产业发展集群化。高度重视园区建设，加快百里工业经济带及隆尧东方食品城、清河羊绒科技园、宁晋单晶硅园区、平乡自行车工业园、临西轴承工业园、沙河金百家玻璃等园区和重点镇工业集中区建设步伐，发挥骨干企业的核心带动作用，引导各类生产要素向园区聚集，促进产业升级和集群集约发展。

　　（二）以促进农民增收为核心，扎实推进新农村建设。要围绕农业发展、农民增收，优化农业结构，发展现代农业，大力扶持龙头企业，完善农村基础设施和公共服务体系，推进农业产业化经营，加快农业大市向农业强市转变。

　　调整农业结构。在继续大力发展粮食、棉花生产，稳定面积，提高单产，增加总产量的基础上，进一步发展壮大畜牧、蔬菜、林果三大主导产业，重点抓好生猪、奶牛、肉鸡、蛋鸡四大养殖基地建设，促进规模化和标准化。加大设施蔬菜、优质果品等种植面积，增加市场供给。推进农业标准化生产，提高农产品的质量安全水平。推广林粮、棉菜间作套种等复合经营模式，抓好农产品生产标准化管理和绿色食品生产，提高农业综合效益。

　　培育农业产业化龙头。把发展现代农业与发展农产品加工结合起来，下大力抓好方便食品、肉类加工、粮食深加工等，新增市级农业产业化龙头企业15家以上，带动农产品加工转化增值，增加农业产出效益。同时，扶持从事农产品流通的经纪人及农民专业合作组织，不断提高农户发展生产和进入市场的组织化程度。加快太行山区综合开发，积极发展生态节水农业。

　　完善农村基础设施和公共服务体系。加强对基本农田的保护，切实搞好农田水利基础设施建设。全年完成水利建设投资2.4亿元，重点抓好农村供水、水库加固、防洪、节水灌溉、生态恢复等工程建设。加强林业生态建设，全年完成造林绿化33万亩。抓好县、乡公路改造养护。积极推广农村有线电视入户业务，为农民提供文化娱乐和信息服务。

　　拓宽农民增收渠道。加强农村市场流通体系建设，壮大和提升农村二三产业，增强县域经济实力。继续实施“阳光工程”，加强对农村劳动力的职业技能培训，提高农民转移就业能力，实现农村劳动力向非农产业和城镇转移21.2万人（次）。进一步加大扶贫开发力度，减少贫困人口数量。

　　（三）加快城镇化步伐，提高区域经济竞争力。城镇化是实现工业化、现代化的必然要求，也是实现城乡发展一体化，从根本上解决“三农”问题的主要途径。

　　完善城镇规划体系，优化城镇空间布局。要顺应城镇化发展趋势，高标准、高起点编制好市、县和重点镇三级总体发展规划。要推进城镇化与工业化良性互动，既通过工业化推动城镇化，又通过城镇化提高对工业化的承载能力。各县（市）要根据资源禀赋、特色优势，在城镇规划建设中突出搞好产业布局，完善功能分区，土地集约利用，资源优化配置，使工业向园区集中，居民向社区集中。要搞好城镇集中供水排水、集中供热供气、集中排污治污等各类管网的综合布局、长远设计，避免出现“今天建、明天拆”的浪费现象。健全规划公众参与制度，维护城镇发展规划的严肃性，加大规划监察力度，严厉查处各类违法建设。培育发展城镇体系，推进以邢台主城区为中心，以清河、宁晋为次中心，以沙河、南和、任县、内邱、皇寺为卫星城，以百里工业经济带、百里太行旅游带为连绵区，以重点镇为辐射点的城镇化网络建设。通过中心城区发展，带动周边城镇的发展，推动城市产业、基础设施、公共服务、城市文明向农村延伸辐射，促进城乡之间良性互动、协调发展。

　　发展壮大市本级经济，提高中心城市辐射带动能力。拉大城市空间，搞好产业布局。坚持“产业立市”，围绕中心城市区域内的冶金机械装备制造、煤化工、硅材料等优势产业，优化产业布局，推进集约、集成发展；实施骨干龙头企业“再造工程”，鼓励和支持市属企业扩张规模，优化结构，提升产业，增加效益，扩大税源；把高开区作为带动市本级经济发展的引擎，加大开放招商力度，承接产业转移，引进、建设一批高新技术产业项目，尽快把高开区建设成为创业环境宽松、产业聚集度高、充满发展活力的经济新区；依托桥东、桥西区现有产业基础，强化配套服务，努力建设成为现代服务业的集中区、生产要素的集散区。

　　加大城镇基础设施建设力度，提高发展承载能力。把服务经济发展、改善市民生存环境、提高市民生活质量作为城镇建设的出发点和落脚点，加大城镇基础设施建设力度，提高建设水平，发挥中心城市的示范带动作用。市区全力实施“810工程”，大力开展城镇面貌“每年一大步、三年大变样”活动，今年实施8大类39个重点项目。七里河综合治理完成13公里河道治理、17公里景观道路建设等工程，开工建设邢州路和南水北调干渠两座景观桥。新建、改造百泉大道、邢州路南延等14条道路。抓好中兴东大街旧区改造、东大街区片改造、桥东区“小吃城”、北大郭区片综合改造开发等项目工程建设。启动改造10个“城中村”，推进旧城改造。改造提高广场游园17个，新建金牛中兴广场等街头游园10个。治理紫金泉东街、五四街北段等小街小巷13条。完成主要街道的广告、牌匾治理。实施夜景亮化工程。完成拆违拆迁100万平方米以上。抓好市生活垃圾、医疗垃圾、城市污水处理项目建设和运行，提高城市垃圾和污水处理能力。加快推进一批城镇基础设施建设，清河、宁晋、南宫污水处理厂上半年投入运行，内邱、临城、新河、威县、隆尧、巨鹿等县城污水处理厂年底建成投运。

　　加快交通项目建设，积极构筑西联东出嗤ㄊ嗯ΑＨ杲煌ɑ∩枋┙ㄉ杓苹瓿赏蹲?0亿元，重点推进投资4.4亿元高速公路项目，继续加快邢临、青银高速公路连接线建设和邢港、环城高速公路前期工作，大广、邢汾高速公路开工建设。

　　（四）大力推进服务业发展，优化三次产业结构。突出发展现代服务业。优先发展现代物流、信息服务、研发设计、商务服务、金融保险等生产性服务业，为加速工业化进程提供配套服务。紧紧抓住高速公路、铁路等基础设施建设的重要机遇，谋划建设大型物流园区、专业市场和内陆港、铁路新货场等三产项目，加强南北方向的经济合作与要素交流，畅通东西方向的物资运输，逐步把邢台打造成沟通南北、西联东出的物流枢纽。同时，积极拓展健身、娱乐、休闲等文化服务领域，发展养老、托幼、社区服务等新兴服务业，大力发展投资咨询、资产评估等咨询服务业以及会展、法律、会计等中介服务业。

　　巩固提升传统服务业。采取优惠政策，发挥资源和特色产业优势，鼓励和扶持一批销售生产资料、生活资料的大型专业批发市场；适应对外开放水平的提高和群众消费需求的提升，引进和建设一批大型商场和高档接待场所，争取全国商业百强入驻我市，加快五星级万峰大酒店建设进度，提高接待水平；围绕方便普通市民购物休闲，增加商业网点和健身娱乐场所，增加面向群众消费、物美价廉的地方特色餐饮网点，促进我市传统服务业优化升级、业态创新，不断提高发展水平。

　　做优做强旅游业。以创建国家优秀旅游城市为目标，集中力量打造“守敬故里、山水太行、开元古刹、七夕之源、抗大圣地”等旅游品牌，抓好重点景区和重点旅游线路建设，开发具有地方特色的精品景区，加快构建百里太行旅游带。

　　（五）推进改革开放，增强发展活力。以纪念改革开放30周年为契机，着眼于完善社会主义市场经济体制，增强经济发展活力，推进重点领域和关键环节的改革，加快对外开放步伐。

　　积极推进改革。以国有资本调整和国有企业改制重组为重点，加快国有工业企业和市直商贸、物资、供销等企业改革步伐。继续推进财政体制改革，理顺规范财政收入分配关系，实施新的激励性财政体制；实施综合治税，通过加快企业主辅分离等有效手段，强化税收征管，优化财政收入结构，进一步提高财政收入占GDP的比重、一般预算收入占全部财政收入的比重、税收占财政收入的比重。深化投融资体制改革，推进“四资”整合运作。积极推进事业单位、农村综合改革、商贸流通领域等各项改革。

　　扩大开放招商。抓住国际国内产业加快转移和京津冀都市圈建设的机遇，靠开放深化改革，靠开放引进项目，靠开放吸引人才。明确招商方向，以重点企业和园区为招商主体，加强与国内外大集团、大公司的联系，积极引进战略投资者。注重招商成效，加大已签约项目落实力度，突出抓好利用外资1000万美元以上项目和利用内资5000万元以上项目，努力提高对接的成功率、合同的履约率和资金的到位率。调整出口商品结构，努力扩大对外贸易，鼓励机电产品和高新技术产品出口，提高纺织服装、羊绒、玻璃等传统产业出口商品质量和档次；培育壮大出口龙头企业和重点出口基地。争取年内建成邢台海关；争取有两家以上全国性股份制银行在我市建立分支机构；加强与深交所、上交所的联系，争取完成3家企业上市工作。

　　增强创新能力。发挥企业的创新主体作用，鼓励大中型企业增加科技投入和建立研发机构，支持中小企业开展技术创新和产品研发，着力提升主导产业的技术水平和产品的科技含量。实施名牌战略，打造一批具有自主知识产权和较强市场竞争力的知名品牌，帮助永进电缆、远大阀门和晶牛微晶玻璃等申请中国驰名商标，支持邢机轧辊、晶澳太阳能电池、蓝鸟沙发申请中国名牌产品，力争新增省以上知名品牌10个以上。加强人才队伍建设，创新人才激励保障机制，吸引各类人才来我市发展。

　　培育更多的市场主体。创新和完善一系列优惠政策，支持和鼓励更多的人自主创业，扶持培养一批发展前景好、成本比较小的中小企业，以创业带动就业。要围绕创业搞好服务，年内争取每个县（市、区）至少建成一个功能完善的中小企业创业辅导基地，筹集担保资本金1亿元，为中小企业提供贷款担保5亿元以上。

　　大力优化发展环境。要支持和鼓励全民创业，扩大开放招商，必须继续优化创业环境和氛围。各级政府部门要进一步减少审批环节，提高办事效率。加大对企业发展环境的综合整治力度，对优秀企业家和外来客商要尊重和保护，提高创业者的社会地位，维护投资者的合法权益。加强信用环境建设，优化金融生态环境。加大对优化发展环境的监督和奖惩力度，树立优化环境的正面典型，对影响和破坏发展环境的反面典型及时发现和处理。

　　（六）狠抓节能减排，增强可持续发展能力。坚持发展与治理兼顾、疏导与防堵结合、增优与汰劣并重，工程性措施与制度性约束并举，从最紧迫、最关键的环节抓起，打好节能减排攻坚战、持久战。

　　实施重点突破。对列入省节能减排“双三十”的3个县和4家重点企业，定出阶段性目标，确保按期并力争提前完成考核任务；对列入国家、省重点考核的16家企业和市选定的14家重点耗能企业，加强监测与考核；对71家国、省控重点污染企业加强监管，确保完成主要污染物排放量削减任务；对污染严重、群众反映强烈的工业企业坚决实施停产治理。同时，对市定的30个重点工业集中区、30家重点企业，进行节能减排市级考核，3年内完不成任务的县（市、区）政府主要领导要引咎辞职，国有企业法人代表就地免职，民营企业依法责令停产整治。

　　淘汰落后产能。落实好我市“十一五”期间淘汰落后产能计划，确保淘汰落后装备按计划如期退出市场；按照国务院和省政府要求，年内依法依规关闭60%的立窑小水泥企业，两年内全部关闭；加大煤矿和非煤矿山整合力度，对不符合国家产业政策的小玻璃、小造纸、小板材、小纺织等三年内依法全部取缔。

　　发展循环经济。加快建设科学合理的能源资源利用体系，提高能源资源利用效率和保障能力；加大对循环经济的政策扶持力度，在项目审批、土地供应等方面给予重点扶持。在煤炭、钢铁、建材、电力、化工等行业推进循环经济试点工作，今年循环经济企业力争发展到20家以上。

　　加强生态环境保护。巩固取缔“四小”成果，实施市区大气综合治理工程，拆除燃煤锅炉100台，改造50家餐饮单位使用天然气、煤气等清洁能源，市区空气质量二级及好于二级天数达到320天以上。实施生态环境建设工程，重点抓好西部山区的生态环境保护，严格控制片麻岩和红板石开采中对山体植被的破坏，让邢台的天更蓝、山更绿、空气更清新。

　　（七）加快发展社会事业，努力改善民生。在发展经济的同时，更加注重社会建设，努力保障和改善民生，促进社会公平正义，推动邢台和谐发展。

　　优先发展教育。加大教育投入，全面提升办学水平和教育质量，办好各级各类教育。整合教育资源，搞好新一轮中小学布局调整，建设一批标准化学校。提高农村家庭经济困难寄宿生生活补助标准，免除义务教育阶段学生学杂费的范围由农村扩大到城市。新建、改造85所中小学，让孩子上得起学，争取让孩子不必择校上好学，做到学有所教。实施温暖工程，争取全市农村中小学今年冬季全部用上暖气。全面实施素质教育，推进教育改革创新，切实减轻中小学生的课业负担。加强教师队伍特别是农村教师队伍建设，完善和落实教师工资、津补贴制度。做大做强职业教育，加强职业教育基础能力建设，深化职业教育管理、办学、投入等体制改革，筹建职教园区，打造多层次、多体制办学模式，培养高素质技能型人才。深入实施高校质量工程，优化学科专业结构，提高办学水平。

　　积极扩大就业。加强就业和创业培训，鼓励自谋职业和自主创业，以创业带动就业。完善公共就业服务体系，促进形成城乡劳动者平等就业制度。加强高校毕业生就业指导和服务，做好退役军人安置工作。落实困难群众就业援助制度，提高零就业家庭就业稳定性。全市城镇新增就业4万人，城镇下岗失业人员实现再就业1.6万人。

　　健全社会保障体系。抓好社会保险扩面，启动城镇居民医疗保险，逐步实现各类企业应参尽参。城镇低保做到应保尽保，农村低保面扩大到3.5%以上，低保对象达到20万人以上。对廉租住房户做到应保尽保，落实资金2500万元，补贴经济适用住房户1000户。落实五保供养政策，每县至少建成一所星级敬老院，提高集中供养率。建设市儿童福利院，积极救助困难儿童。高度重视社会福利和残疾人事业，鼓励社会力量参与公益事业。

　　加快文化事业和文化产业发展。加大投入力度，重点抓好市图书馆、市广电中心、市博物馆、市文化活动中心等公共文化服务设施建设，扎实推进社区、居民小区、乡镇文化站和村文化室等惠民工程，全国爱国主义教育示范基地全部向社会免费开放，满足广大人民群众参加文化活动和接受教育的需要。积极培育发展文化产业，引导有实力的企业投资文化产业，鼓励和扶持个人开发创办文化实体。

　　提高医疗卫生水平。全面实行新农合制度，参合率达到85%以上，扩大城镇职工基本医疗保险覆盖面；完善公共卫生服务体系，推进公共卫生工作的规范化管理。进一步健全卫生应急管理和协调机制，有效应对各类重大传染病疫情和突发公共卫生事件。以完善农村卫生机构功能和提高服务能力为核心，以乡镇卫生院标准化建设为重点，健全县、乡、村三级医疗卫生服务网络，不断提高基层卫生服务条件和能力，要让群众看得起病，争取让群众不出远门看得好病。到年底，全市80%的乡镇卫生院要达到标准化建设要求，农村卫生室覆盖率达到95%以上。改善医疗卫生服务，创造良好就医环境。加强妇幼保健体系建设，提高妇女儿童健康水平。加强食品药品市场监管，保证群众食品用药安全。

　　加强人口和计划生育工作。继续加大计划生育工作的宣传力度，提高全社会对计划生育工作重要性的认识，巩固集中整治成果，毫不放松地做好抓基层、打基础工作。稳定低生育水平，提高出生人口素质，综合治理出生人口性别比偏高问题。全面实施计划生育家庭奖励和特别扶助制度，提高奖励扶助标准。加强流动人口计划生育服务管理。

　　维护社会稳定。认真解决涉及群众切身利益的热点难点问题，把矛盾化解解决在萌芽状态。千方百计保障市场供应，努力保持市场物价基本稳定。加强社会治安综合治理，完善治安防控体系，依法严厉打击各种犯罪活动，促进平安邢台建设。完善应急救援体系，提高预防和处置突发公共事件的能力。加强安全生产监督和管理，严防重特大安全事故发生。深入开展双拥共建活动，支持国防后备力量建设。做好民族宗教、外事侨务、人防、气象、地震、档案、地方志等各项工作，促进社会全面进步。

　　（八）加强政府自身建设，不断提高执政为民的能力。继续深入开展解放思想大讨论活动，积极转变政府职能，切实改进工作作风，不断提高行政效能，努力建设人民满意的政府。

　　努力提高贯彻落实科学发展观的能力。我们要认真学习十七大精神，不断加深对科学发展观精神实质的理解和把握，真正在转变经济发展方式、调整优化结构上下功夫，真正把科学发展观落实到想问题、做决策、抓工作的全过程。注重发挥市场在资源配置中的基础性作用，善于用市场经济的思路、理念和办法解决发展中的困难和问题。

　　加快转变政府职能。积极推进政企分开、政资分开、政事分开，解决政府职能越位、缺位和错位问题。特别注意凡是用市场办法能解决的问题，政府就不要多管。继续深化行政审批制度改革，做到多服务、少检查，多指导、少收费，多教育、少罚款。坚持依法行政，严格按照法定权限和程序行使职权、履行职责。进一步健全考核机制和行政问责制，加大对滥用权力和失职、渎职行为的追究力度，改变有令不行、有禁不止现象。

　　切实改进工作作风。要树立勤政为民、务实高效、奋发有为、开拓创新的工作理念，扎扎实实推进各项工作开展。压缩会议文件，减少不必要的应酬，集中精力抓改革、谋发展、保稳定、促和谐。以打造阳光政府、效率邢台为核心，扎实推进机关效能建设，切实提升执行力。在市政府部门推行“局长创优”、“科长创新”活动，切实提高办事效率。深入开展行风政风民主评议活动，大力纠正行业和部门不正之风，严肃整治损害群众利益和破坏投资环境的行为。建立健全督查落实机制，强调落实的刚性，务求实效。

　　进一步加强廉政建设。坚持标本兼治、综合治理、惩防并举、注重预防的方针，加强反腐倡廉教育和公务员职业操守教育，自觉加强从政道德修养。倡导艰苦奋斗的优良传统，厉行节约，勤俭办事，努力降低行政成本。深入推进政务公开，完善对权力的监督制约机制，坚持用制度管权、管事、管人。自觉接受人大的法律监督、工作监督、政协的民主监督和广大群众的社会监督。严肃查处各类违法违纪案件，依法严惩腐败分子。

　　各位代表！

　　回顾过去的五年，我们取得了巨大成就。清醒地分析现状，我们面临着严峻挑战和前所未有的机遇。展望未来，我们充满自信和勇气。让我们在省委、省政府和市委的领导下，高举中国特色社会主义伟大旗帜，以邓小平理论和“三个代表”重要思想为指导，深入贯彻落实科学发展观，解放思想，求真务实，锐意进取，埋头苦干，为实现邢台经济社会又好又快的发展而努力奋斗！
